# Improved anticancer activity of betulinic acid on breast cancer through a grafted copolymer-based micelles system

**DOI:** 10.1080/10717544.2021.1979125

**Published:** 2021-09-25

**Authors:** Xueju Qi, Cong Gao, Chuanjin Yin, Junting Fan, Xiaochen Wu, Chuanlong Guo

**Affiliations:** a Department of Pharmacy, College of Chemical Engineering, Qingdao University of Science and Technology, Qingdao, China; b Affiliated Hospital of Shandong Academy of Medical Sciences, The Third Affiliated Hospital of Shandong First Medical University, Jinan, China; c Department of Pharmaceutical Analysis, School of Pharmacy, Nanjing Medical University, Nanjing, China

**Keywords:** Betulinic acid, Soluplus, breast cancer, DNA damage, angiogenesis

## Abstract

Betulinic acid (3β-Hydroxy-20(29)-lupaene-28-oic acid, BA) has excellent anti-cancer activity but poor solubility and low bioavailability. To improve the antitumor activity of BA, a polyvinyl caprolactam–polyvinyl acetate–polyethylene glycol (PVCL–PVA–PEG) graft copolymer (Soluplus) encapsulated BA micelle (Soluplus-BA) was fabricated. The Soluplus-BA micelles presented a mean size of 54.77 ± 1.26 nm and a polydispersity index (PDI) of 0.083. The MTT assay results showed that Soluplus-BA micelles increased the inhibitory effect of BA on MDA-MB-231 cells, mainly due to the enhanced accumulation of reactive oxygen species (ROS) and the destruction of mitochondrial membrane potential (MMP). Soluplus-BA micelles induced the DNA double-strand breaks (DSBs) as the γH2AX foci increased. Moreover, Soluplus-BA also inhibited the tube formation and migration of human umbilical vein endothelial cells (HUVECs), and inhibited the neovascularization of the chicken chorioallantoic membrane (CAM). This angiogenesis inhibitory effect may be accomplished by regulating the HIF-1/VEGF-FAK signaling pathway. The *in vivo* study confirmed the improved anti-tumor effect of Soluplus-BA and its inhibitory effect on angiogenesis, demonstrating the possibility of Soluplus-BA as an effective anti-breast cancer drug delivery system.

## 1. Introduction

According to statistics, there are ∼19.3 million new cancer cases and nearly 10 million cancer deaths worldwide in 2020 (Moreira et al. 2021). Female breast cancer has surpassed lung cancer as the most common tumor, with ∼2.3 million new cases (11.7%) (Siegel et al. 2020; Sung et al. 2021). Breast cancer is a malignant tumor that occurs with multiple genes involved in multiple steps. The current drug treatment of breast cancer mainly includes chemotherapy and endocrine (Azim and Ibrahim 2014; DeSantis et al. 2019). Breast cancer is often accompanied by a high recurrence rate, thus it is very important to find specific drugs to treat it.

Betulinic acid (3β-Hydroxy-20(29)-lupaene-28-oic acid, BA) is a pentacyclic triterpene that can be obtained from many fruits, plants and vegetables (Rios and Manez 2018). BA has a variety of biological activities including anti-tumor, anti-viral, anti-inflammatory and anti-oxidant activities (Wang et al. 2017; Cai et al. 2018). In recent years, the anti-tumor activity of BA has received widespread attention, as it can inhibit the growth of various tumor cells, such as melanoma, ovarian cancer, and lung cancer (Jiao et al. 2019; Zheng et al. 2019; Liao et al. 2020). However, BA is insufficient in some physicochemical and pharmaceutical properties, for example, the poor water solubility greatly limits its clinical development. To alleviate the poor water solubility and bioavailability of BA, suitable BA delivery systems to increase its biological activity has become a research direction (Dutta et al. 2019; Wang et al. 2019).

Soluplus is an amphiphilic polyethylene caprolactam–vinyl acetate–polyethylene glycol (PVCL–PVA–PEG) graft copolymer that can self-assemble in solution to form micelles and encapsulate drugs with poor solubility (Hou et al. 2016; Zhu et al. 2019). The carbonyl groups of Soluplus can form hydrogen bonds with the hydroxyl groups of the drug molecules, thereby increasing the drug solubility and stability. There are studies showing Soluplus as a carrier to deliver drugs and treat tumors and corneal diseases, confirming its high potential as a drug delivery carrier (Guo et al. 2016; Nam et al. 2017).

In this study, we successfully prepared BA-loaded Soluplus micelles (Soluplus-BA) with high encapsulation efficiency and stability. The Soluplus-BA micelles can significantly improve the growth inhibitory effect of BA on breast cancer MDA-MB-231 cells. The effect and mechanism of using Soluplus as a carrier to deliver BA in the treatment of breast cancer was also investigated.

## 2. Materials and methods

### 2.1. Materials

PVCL-PVA-PEG (Soluplus) was purchased from BASF Ltd. (Shanghai, China). Betulinic acid (BA) was purchased from Dalian Meilun Biology Technology Co., Ltd. (Liaoning, China). Dulbecco’s Modified Eagle’s Medium (DMEM) medium was obtained from Hyclone (Logan, UT, USA). Fetal bovine serum (FBS) was obtained from ExCell Bio (Shanghai, China). Antibodies against γH2AX and GAPDH were purchased from Cell Signaling Technology (Beverly, MA, USA).

### 2.2. Animals

For animal studies, Kunming mice were obtained from Huafukang (Beijing, China). The animal care and procedures were conducted according to the Principles of Laboratory Animal Care. The animal study was approved by the Qingdao University of Science and Technology Ethics Committee for Animal Experimentation (approval document no. 2017-1, Qingdao, China).

### 2.3. Preparation of the Soluplus micelles

Soluplus-BA were prepared by a thin film hydration method as previously reported (Guo et al. 2015b). Briefly, BA (1 mg) and Soluplus (with different Soluplus/BA weight ratios) were dissolved in ethanol. The solvent was slowly evaporated using a rotary evaporator under reduced pressure at 40 °C until a dry film was formed on the inner wall of the flask. Phosphate buffer solution (PBS) was added, and the flask was rotated under normal pressure for 10 min by using a rotary evaporator at 120 rpm in a 30 °C water bath to hydrate the dried film with PBS to obtain a micelle dispersion. The micelles were filtered through a 0.22 μm filter to discard the unencapsulated BA. The encapsulation efficiency of BA in Soluplus-BA micelles was analyzed using high-performance liquid chromatography (HPLC).

### 2.4. Characterization of Soluplus-BA micelles

The particle size, zeta potential, and polydispersity index (PDI) of micelles were analyzed using a Zetasizer (Malvern MS2000, UK). The morphology of micelles was examined by transmission electron microscopy (TEM) using a JEM-1200EX microscope (JEOL Ltd., Tokyo, Japan), as previously reported.

### 2.5. Cellular uptake assay

Coumarin 6 is a common tool molecule used to study the uptake of nano-drugs (Guo, Cui, et al. 2015). In this study, coumarin 6-loaded Soluplus (Soluplus-Cou6) micelles were successfully prepared to study the cellar uptake of Soluplus delivery system. The localization of Soluplus micelles in MDA-MB-231 cells has also been studied by fluorescently labeling lysosomes and mitochondria in MDA-MB-231 cells. Briefly, MDA-MB-231 cells were stained by 100 nM Lyso-Tracker and 1 µM ER-Tracker for 20 min, cells were then treated with Soluplus-cou6 micelles for 15 min. After 3-times of washes by PBS, cells were detected and photographed by fluorescence microscope.

### 2.6. *In vitro* anticancer activity assay

#### 2.6.1. MTT assay

MDA-MB-231 cells were seeded in 96-well plates and incubated overnight. Cells were treated with Soluplus alone, variety of Soluplus-BA or free BA for 48 h. After the treatments, 100 µl of MTT (0.5 mg/ml) was added in the plates and incubated for 4 h. The supernatant was removed, 150 µl of DMSO was added, and after shaking for 10 minutes, the absorbance at 490 nm was measured and the inhibition rate was calculated.

#### 2.6.2. Colony formation assay

MDA-MB-231 cells were seeded in 6-well plate at a density of 1000 cells/well. After 24 h of incubation, cells were treated with Soluplus-BA or free BA for 48 h. The medicated culture medium was replaced with fresh culture medium, and the cultured for another 10 days. The cells were fixed with 4% paraformaldehyde and stained with crystal violet and counted.

#### 2.6.3. Wound healing assay

MDA-MB-231 cells were seeded in 24-well plate at a density of 2 × 10^5^/well. When the cells were 90% confluent, cells were scratched with a 200 µl pipette tip. The culture medium was aspirated and fresh medium containing Soluplus-BA or free BA was added. The wound healing was observed and photographed at 0, 12 h and 24 h respectively.

#### 2.6.4. Immunofluorescence assay

MDA-MB-231 cells were seeded in 48-well plate and incubated for 24 h. Cells were treated with Soluplus-BA or free BA for 48 h. Cells were then fixed with 4% paraformaldehyde and blocked with 5% BSA for 1 h at room temperature. Cells were incubated with γH2AX antibody overnight, washed with TBS-T for 3 times and incubated with fluorescently labeled secondary antibody for 1 h. After 3-times of washes, the nucleus were stained with DAPI and then cells were observed with a fluorescence microscope and photographed.

#### 2.6.5. Ros generation and JC-1 staining assay

MDA-MB-231 cells were seeded in 24-well plate and incubated overnight. Cells were treated with Soluplus-BA or free BA for 48 h. Cells were stained with DCFH-DA/JC-1 for 20/30 min. After 3-times of washes, cells were photographed under a fluorescence microscope.

### 2.7. Detection of angiogenesis

#### 2.7.1. Tube formation assay

HUVEC cells were used to detect the effect of BA on angiogenesis. Briefly, 50 µl of Matrigel^TM^ was pipetted into 96-well plates and incubated overnight at 37 °C. HUVEC cells with Soluplus-BA micelles or free BA were seeded onto the Matrigel. After 6 h of incubation, cells were photographed under an inverted contrast phase light microscope.

#### 2.7.2. Chick chorioallantoic membrane (CAM) assay

The chick embryo allantoic membrane method was used to detect the inhibitory effect of Soluplus-BA on angiogenesis. Briefly, embryonic eggs were incubated in a humidified (65–70%) incubator for 5 days. Then the air sac was opened to expose the air chamber, and the sterile filter paper containing the drugs was putted into the CAM and incubated for another 24 h. After incubation, CAM was examined under microscope and photographed.

### 2.8. Western blot analysis

Western blot analysis was performed as previously reported (Guo et al. 2020).

### 2.9. *In vivo* antitumor analysis

4T1 cells were cultured and collected, 2 × 10^6^/ml cells were injected into the armpit of the mice. When tumors were grown to 100 mm^3^, the mice were randomly divided into five groups, including control group, Soluplus alone group, free BA group (50 mg/kg), Soluplus-BA (25 mg/kg) and Soluplus-BA (50 mg/kg) group. Mice were performed oral administration every day for 15 consecutive days. After the treatment, mice were sacrificed, and tumors were collected, fixed for immunohistochemistry analysis.

### 2.10. Statistical analysis

Statistical analysis was performed using GraphPad Prism 5.0 (San Diego, CA, USA). The data were presented as mean ± SD. Statistical comparisons were performed using one-way analysis of variance. *p* < 0.05 was considered statistically significant.

## 3. Results and discussion

### 3.1. Preparation and characterization of Soluplus-BA micelles

**Figure 1.**
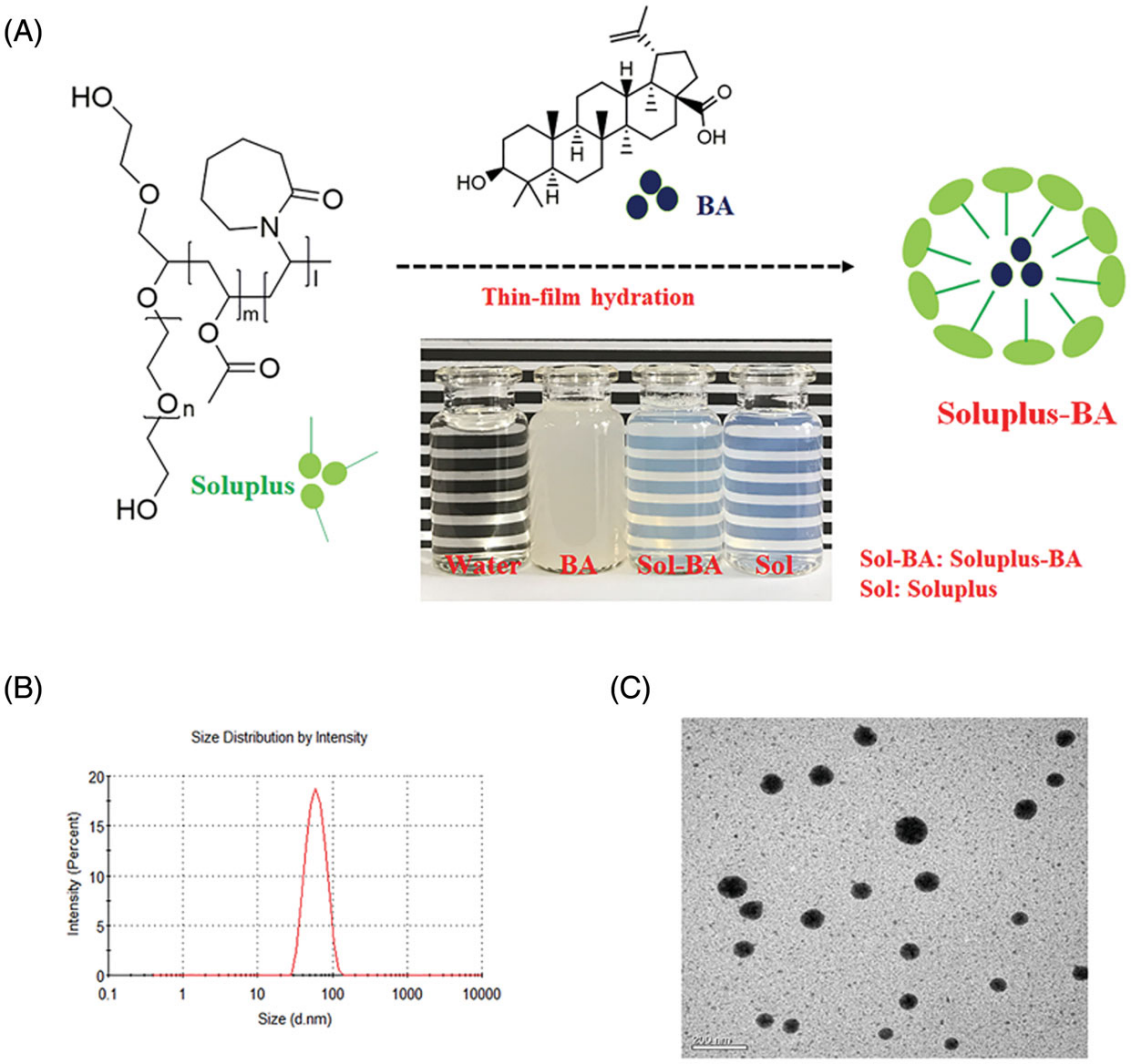
(a) Schematic illustration of self-assembled Soluplus-BA micelles. (b) Micelle size. (c) Transmission electron microscopy (TEM) images of Soluplus-BA (magnification ×400 k, bar =50 nm).

In this study, a thin film dispersion method was employed to prepare Soluplus encapsulated BA micelles (Figure 1(A)). The encapsulation rate increased as the weight ratio of Soluplus/BA increased. For example, when the weight ratio of Soluplus/BA was 10:1, the encapsulation rate of BA was 35%; increasing the Soluplus/BA ratio to 14:1 led to an encapsulation rate of ∼100% (Figure S1). The particle size of the Soluplus-BA micelles in solution was 54.77 ± 1.26 nm, with a polydispersity index (PDI) of 0.083 (Figure 1(B)). The transmission electron microscopy (TEM) images of the Soluplus-BA micelles showed a spherical shape (Figure 1(C)). The zeta potential value of the Soluplus-BA micelles was −1.78 ± 0.78 mV.

The storage stability results showed that Soluplus-BA micelles maintain high stability at both room temperature and 4 °C. In a 9-month experiment, the encapsulation efficiency was maintained >90%, and the particle size was maintained at approximately 56.11 nm, with a PDI of 0.154 (Figure S2).

**Figure 2.**
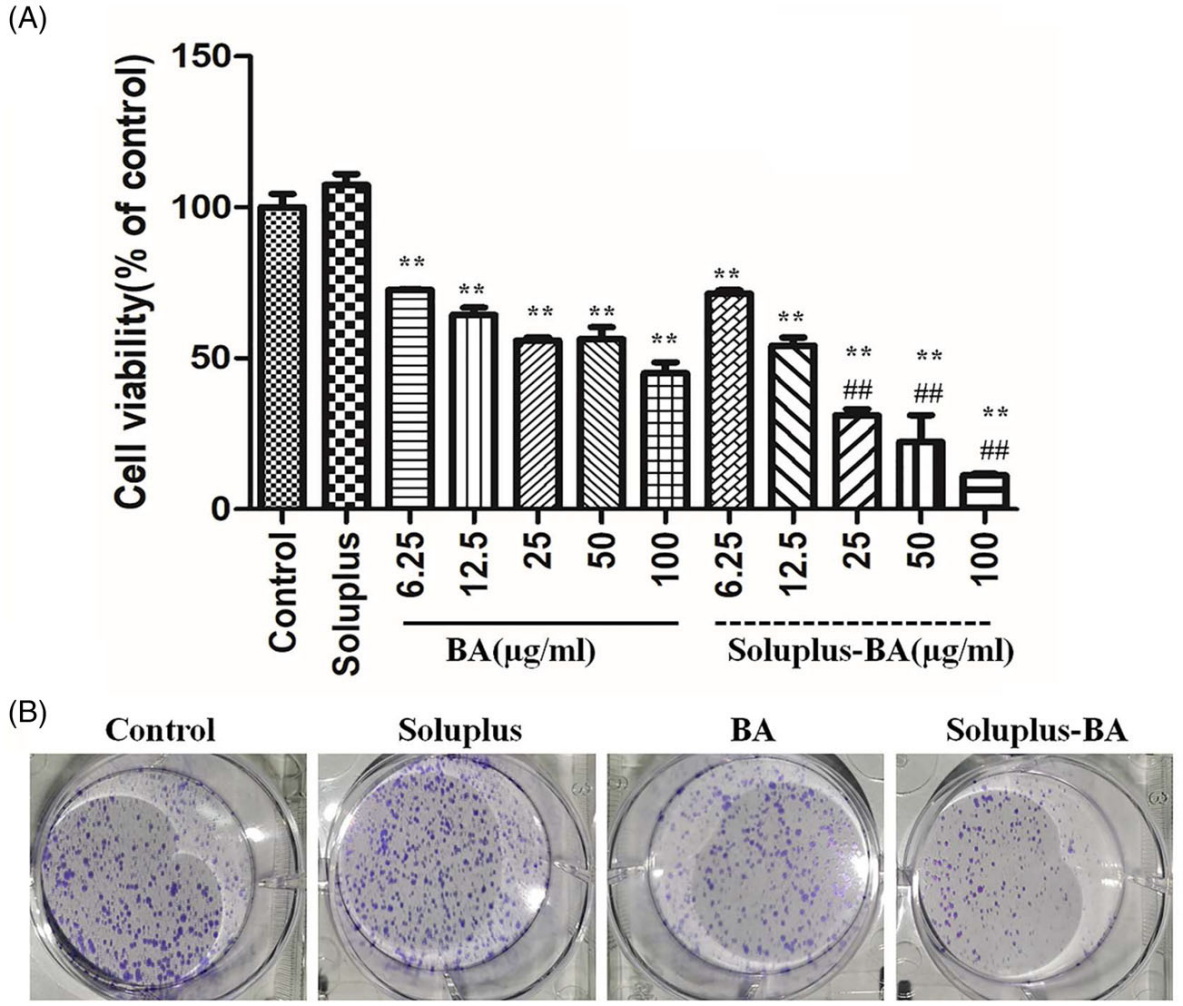
Soluplus-BA micelles inhibit proliferation of breast cancer MDA-MB-231 cells. (A) MDA-MB-231 cells were treated with Soluplus, BA, or Soluplus-BA micelles for 48 h, cell viability was detected by MTT assay. (B) MDA-MB-231 cells (1000 cells/well) were seeded in 6-well plates and treated with BA or Soluplus-BA for 48 h, after changing to fresh culture medium, and continued to culture for 10 days, the cells were stained with crystal violet. **p < 0.05 vs. control group, ##*p* < 0.05 vs. BA group.

The *in vitro* release study suggested that the free-BA and Soluplus-BA micelles had different drug release characteristics (Figure S3). Typically, for a period of 24 h, the free-BA group and Soluplus-BA nano-micelle group exhibited a 62.92% drug release and a 18.02% drug release, respectively. The prolonged drug release characteristics of Soluplus-BA micelles may be attributed to the sustained diffusion of BA entrapped within the cores of Soluplus micelles, similar to the previously reported Soluplus micelles (Dian et al. 2014; Ke et al. 2017). This was probably because BA was well encapsulated in the hydrophobic PVA–PPO core of Soluplus micelles, so it was slowly released from the reservoir through a hydrophilic route.

**Figure 3.**
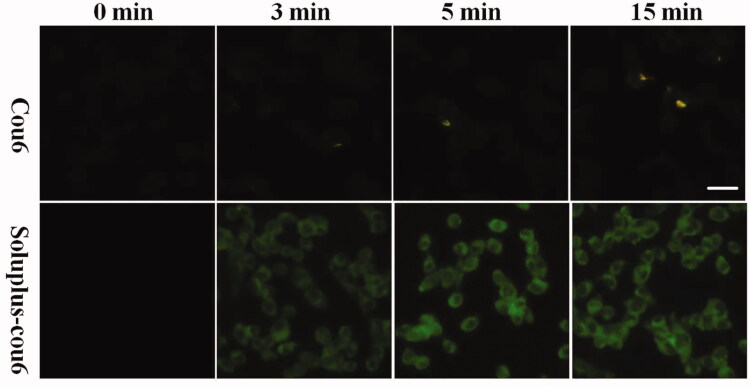
Cell uptake assay. MDA-MB-231 cells were treated with free cou6 and Soluplus-cou6 micelles for 3 min, 5 min, and 15 min, fluorescence was observed and photographed with a fluorescence microscope (Bar = 50 µm).

### 3.2. *In vitro* anti-tumor activity

The *in vitro* anti-tumor activity against human breast cancer was performed by the MTT assay. As shown in Figure 2(A), Soluplus micelles alone showed low cytotoxicity on the MBA-MD-231 cells, indicating that Soluplus micelles are nontoxic drug delivery vehicles. The Soluplus-BA nanomicelle and free-BA groups showed a significant inhibitory effect on the proliferation of MDA-MB-231 cells. The IC_50_ value was 38.81 ± 4.9 µg/mL for free BA and 15.45 ± 3.01 µg/mL for Soluplus-BA micelles. Under the same BA concentration, the inhibition effect of the Soluplus-BA group was more evident, which may due to the improved cell uptake of Soluplus-BA micelles than individual BA. The colony formation results showed similar phenomenon, Soluplus-BA showed a more significant inhibitory effect on the clone formation of MDA-MB-231 than BA (Figure 2(B)).

To evidence the cell update performance of Soluplus-BA, Coumarin 6 was utilized as a probe molecule (Guo, Zhang, et al. 2015). Coumarin 6-loaded Soluplus micelles (Soluplus-cou6) were prepared and applied to detect the uptake and localization of Soluplus micelles in cells. As shown in Figure 3, after 5 min of treatment, the green fluorescence was more significant than that of the free-cou6 groups, indicating that Soluplus-cou6 micelles were quickly taken up by MBA-MB-231.

**Figure 4.**
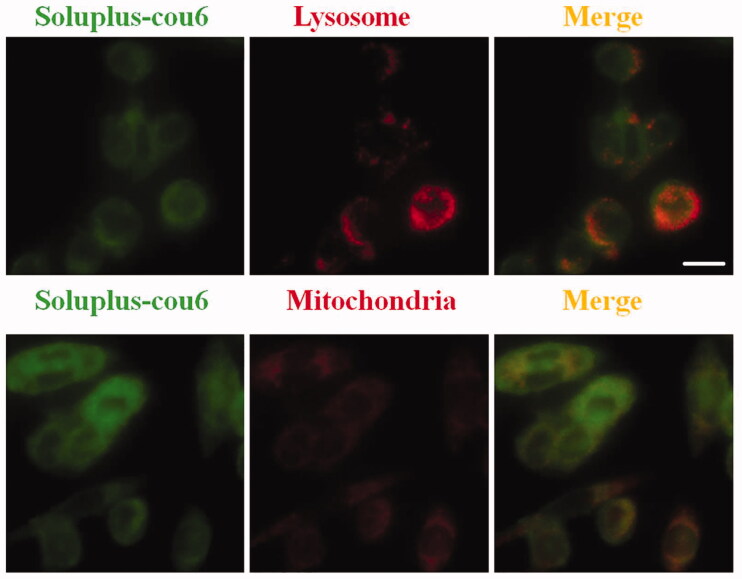
Intracellular trafficking. MDA-MB-231 cells were stained with Lyso-Tracker Red or Mito-Tracker Red for 20 min, after 3-times of washes with PBS, cells were treated with Soluplus-cou6 micelles for 5 min. (A) Colocalization micrographs of micelles with lysosomes. (B) Colocalization micrographs of micelles with mitochondria (Bar = 20 µm).

The co-localization of Soluplus micelles in cells has also been investigated, which may help us to understand the mechanism by which Soluplus-BA inhibitwed the growth of MDA-MB-231 cells. As shown in Figure 4, Soluplus-cou6 micelles can be localized in the lysosomes and mitochondria of MDA-MB-231 cells.

### 3.3. Soluplus-BA micelles induce reactive oxygen species (ROS) generation and mitochondrial membrane potential (MMP) disruption

**Figure 5.**
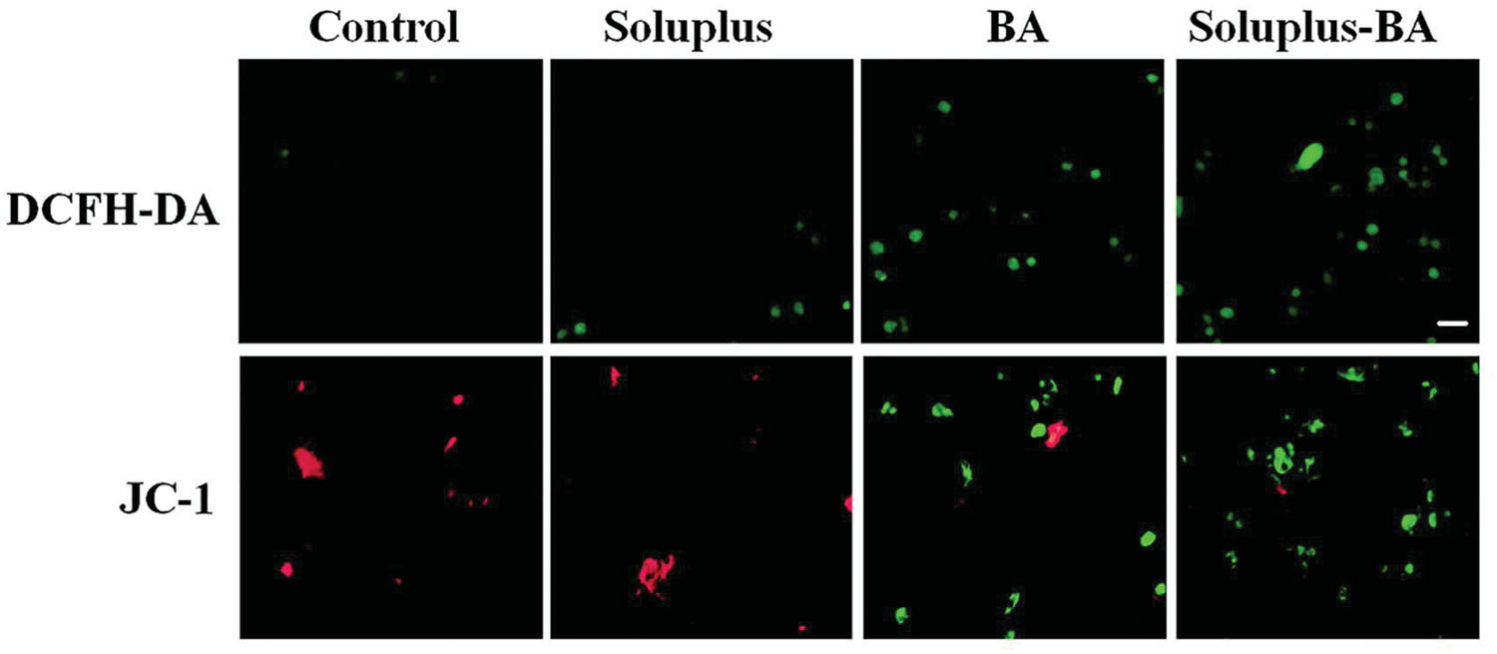
Soluplus-BA micelles induce ROS-mediated mitochondrial apoptosis pathway. MDA-MB-231 cells were treated with Soluplus-BA or BA for 48 h. Cells were stained with DCFH-DA (20 min) or JC-1 (30 min) and photographed under a fluorescence microscope (Bar = 50 µm).

The pathways that regulate cell apoptosis mainly include the mitochondrial-induced apoptosis pathway and the death receptor-mediated apoptosis pathway (Gupta 2001; Chen et al. 2010). In the mitochondrial apoptotic pathway, ROS is an important signal molecule. Studies have found that the accumulation of ROS in cells can induce the destruction of MMP, which ultimately leads to the release of cytochrome c into the cytoplasm, and activates the apoptotic pathway to result in cell death (Fleury et al. 2002; Fang et al. 2016). In this study, dichloro–dihydro–fluorescein diacetate (DCFH-DA) was applied to label intracellular ROS; the results showed that Soluplus-BA micelles significantly induced the formation of ROS in MDA-MB-231 cells. In addition, the JC-1 results showed that Soluplus-BA significantly induced a decrease in MDA-MB-231 mitochondrial membrane potential, indicating that Soluplus-BA can exert anti-tumor activity by inducing ROS-mediated mitochondrial apoptosis (Figure 5).

### 3.4. Soluplus-BA micelles induce DNA double-strand breaks (DSBs) on MDA-MB-231 cells

**Figure 6.**
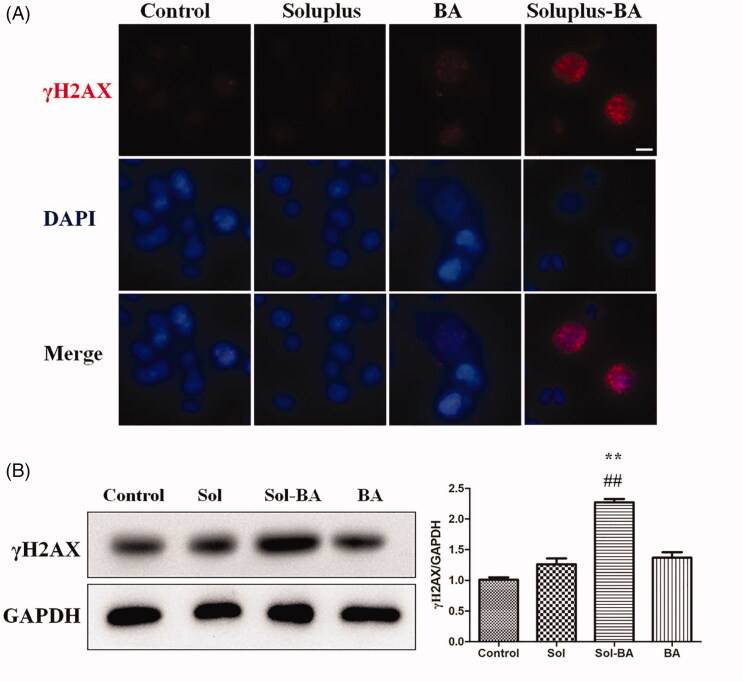
Soluplus-BA micelles induce DNA double-strand breaks. (A) MDA-MB-231 cells were treated with BA or Soluplus-BA micelles for 48 h, the expression of γH2AX was detected by immunofluorescence. (Bar = 50 µm). (B) Expression of γH2AX was analyzed by Western blot. **p < 0.05 vs control group, ##*p* < 0.05 vs. free BA group.

One of the important ways for chemotherapy to exert anti-tumor effects is the induction of DNA damage (McLean et al. 2008). To further clarify the anti-tumor mechanism of Soluplus-BA, immunofluorescence and the western blot were used to detect the effect of Soluplus-BA on the DNA damage of MDA-MB-231 cells. γH2AX is a marker of DNA double-strand damage (Wang et al. 2020). The immunofluorescence results showed that Soluplus-BA can significantly induce γH2AX foci of MDA-MB-231 cells (Figure 6(A)). The western blot results showed similar results, the expression of γH2AX in the Soluplus-BA micellar group was significantly increased comparing to free-BA (Figure 6(B)).

### 3.5. Soluplus-BA micelles inhibit angiogenesis by inhibiting VEGF/FAK pathway

**Figure 7.**
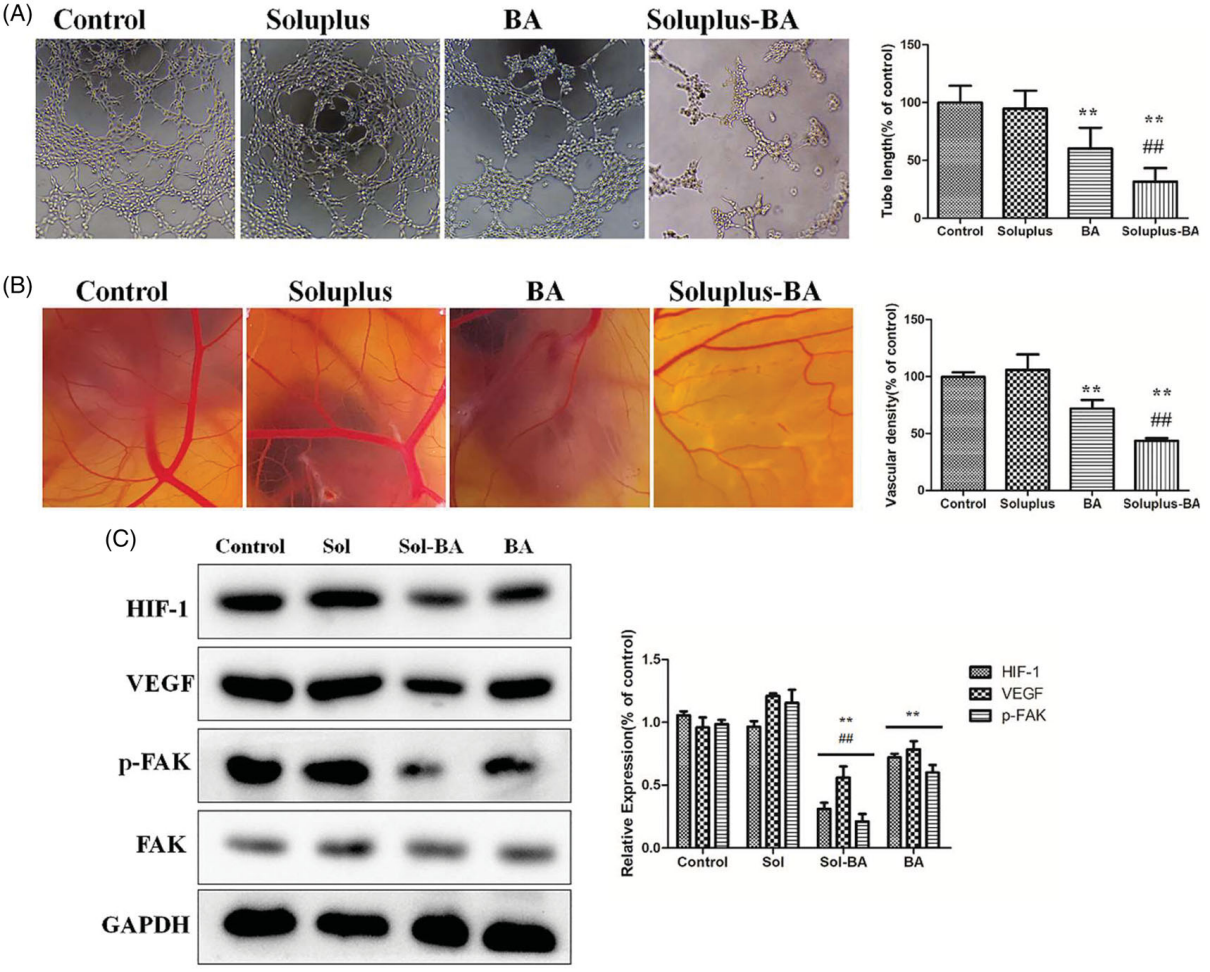
Soluplus-BA micelles inhibit tube formation and ex vivo angiogenesis of CAM. (A) HUVEC cells were seeded in into the Matrigel-coated 96-well plates with Soluplus-BA or BA. After 6 h of incubation, the tube-like networks were photographed (×100). (B) Fertile chicken eggs are placed in the incubator and incubated for 6 days, a small opening was made at the top of eggs and 200 µl of Soluplus-BA micelles or BA was added and incubated for 24 h and photographed. (C) Effect of Soluplus-BA on the expression of HIF-1, VEGF and FAK. **p < 0.05 vs. control group, ##*p* < 0.05 vs. free BA group.

Angiogenesis can provide nutrients for tumors and has an important role in tumor growth, thus the inhibition of angiogenesis is a considerable property of anti-tumor drugs (Sacewicz et al. 2009; Carmeliet and Jain 2011; Schmoll 2011). Soluplus-BA micelles distinctly inhibited the tube formation of human umbilical vein endothelial cells (HUVEC), demonstrating the potential of Soluplus-BA micelles to inhibit angiogenesis *in vitro* (Figure 7(A)). The chicken chorioallantoic membrane (CAM) assay confirmed the anti-angiogenesis effect of Soluplus-BA micelles. As shown in Figure 7(B), comparing with the control group, the capillary density of the Soluplus-BA-treated group was significantly reduced.

**Figure 8.**
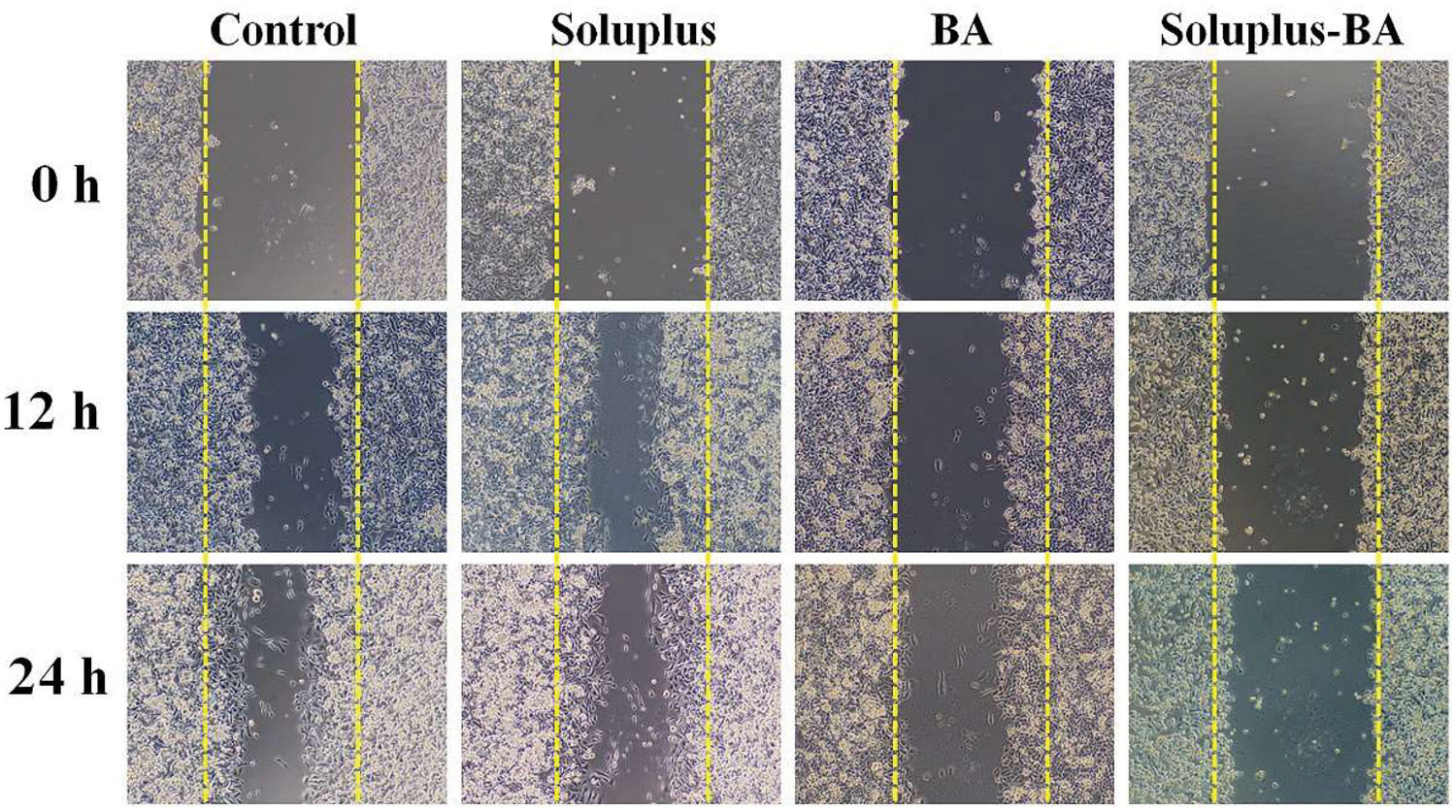
Soluplus-BA micelles inhibit migration. MDA-MB-231 cells were seeded in 24-well plates and incubated at 37 °C. After the cells grew to 90% confluence, they were scratched with a 200 µl pipette tip. The cells were treated with Soluplus-BA or BA, and photographed at 0, 12, and 24 h, respectively.

The high metastatic nature of breast cancer has become a major cause of incurable treatment. New blood vessels provide nutrients for tumors, and the microenvironment formed provides conditions for tumor metastasis (Berz and Wanebo 2011). Tumor metastasis relies on angiogenesis, so inhibiting angiogenesis has positive significance for inhibiting tumor metastasis. In this study, the effect of Soluplus-BA on cell migration was also evaluated by a wound healing assay. As shown in Figure 8, Soluplus-BA micelles clearly inhibited the migration of HUVEC, identifying the ability of Soluplus-BA micelles to inhibit angiogenesis.

Tumor growth depends on angiogenesis and is affected by multiple signaling pathways. Focal adhesion kinase (FAK) is a cytoplasmic tyrosine kinase that plays a basic role in signal transduction mediated by integrins and growth factors, and plays a vital role in cell migration and proliferation (Tavora et al. 2010; Chen et al. 2012). Moreover, tumor cells can release growth factors (e.g., VEGF) into the microenvironment, thereby activating the proliferation of vascular endothelial cells and inducing tumor metastasis (Pang et al. 2011; Bi et al. 2017). Recently, it was discovered that Hypoxia inducible factor-1 (HIF-1) directly regulates the expression of VEGF at the gene level and is an important regulator of malignant tumor-induced angiogenesis (Palazon et al. 2017). In this study, the expression of HIF-1 and VEGF was restrained by free BA as well as Soluplus-BA micelles, while Soluplus-BA micelles showed a more significant inhibitory effect. In addition, BA decreased FAK phosphorylation in HUVEC cells (Figure 7(C)). The angiogenesis inhibitory effect of BA may be accomplished by regulating the HIF-1/VEGF-FAK signaling pathway.

### 3.6. Soluplus-BA micelles inhibit tumor growth *in vivo*

**Figure 9.**
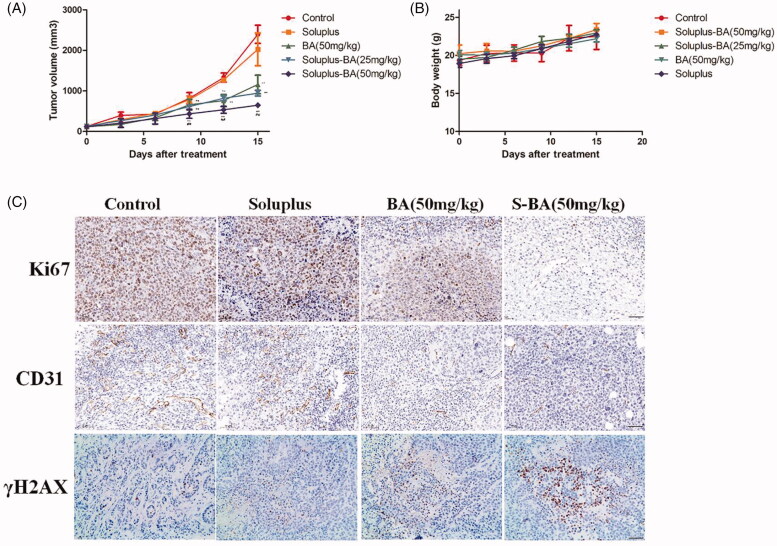
Soluplus-BA micelles inhibit tumor growth in vivo. (A) 4T1 cells were inoculated into the armpits of mice, and the mice were orally administered with PBS, Soluplus, free BA (50 mg/kg), Soluplus-BA (25 mg/kg) and Soluplus-BA (50 mg/kg). Tumor volume was recorded every three days. (B) Body weight. (C) Immunohistochemistry analysis of the expression of Ki67, CD31 and γH2AX (Bar = 200 µm). S-BA: Soluplus-BA micelles. **p < 0.05 vs. control group, ##*p* < 0.05 vs. free BA group.

The above results exhibited that Soluplus-BA micelles inhibit tumor cell proliferation, induce DNA double-strand damage and angiogenesis. To further investigate the anti-tumor activity of Soluplus-BA *in vivo*, a 4T1 mouse tumor model was established and the mice was orally administered Soluplus, free-BA (50 mg/kg), Soluplus-BA (25 mg/kg) and Soluplus-BA (50 mg/kg). After 15 days of continuous administration, the mice were sacrificed. As shown in Figure 9(A), there was no significant difference in the tumors between the control group and the Soluplus group, while the tumor volume of the BA-containing groups was narrowed, and the inhibition effect of the Soluplus-BA nanomicelle group was more significant (Figure 9(A)). Soluplus-BA increased the anti-tumor activity of BA *in vivo*.

We tested the mechanism of Soluplus-BA micelles exerting anti-tumor effects *in vivo*. Chemotherapy drugs such as cyclophosphamide and cisplatin can induce DNA damage and exert anti-tumor effects (Jawad et al. 2010). In recent years, poly adenosine disphosphate–ribose polymerase (PARP) inhibitors, such as olaparib, can inhibit PARP and then induce DNA DSBs to kill Breast Cancer gene (BRCA)-deficient tumors, including breast cancer (Arun et al. 2015; Matulonis et al. 2016). Therefore, inducing DNA damage is a nonnegligible anti-tumor mechanism of drugs. In this study, we detected the expression of γH2AX in the tumor tissues of Soluplus-BA treated mice by immunohistochemistry. As shown in Figure 9(C), the expression of γH2AX in the tumor tissues of the BA group was higher than that of the blank group, and the increase in the Soluplus-BA group was more significant than that of the blank group. In addition, BA induced a decrease in the expression of a cell proliferation marker (i.e., Ki67) (Figure 9(C)). These results show that Soluplus-BA can have an anti-tumor effect *in vivo* by inducing DNA double-strand damage.

On the other hand, breast cancer is a highly metastatic tumor, and inhibiting breast cancer metastasis is also an effective strategy for the treatment of breast cancer (Li et al. 2012). Angiogenesis can provide nutrients for tumors and is the basis of tumor metastasis. In this study, we detected the expression of the angiogenesis marker CD31. A large number of CD31-positive cells were observed in the blank group. BA decreased the cell number, while Soluplus-BA exhibited a more distinct decreasing, demonstrating that Soluplus-BA can inhibit tumor growth by inhibiting angiogenesis.

**Figure 10.**
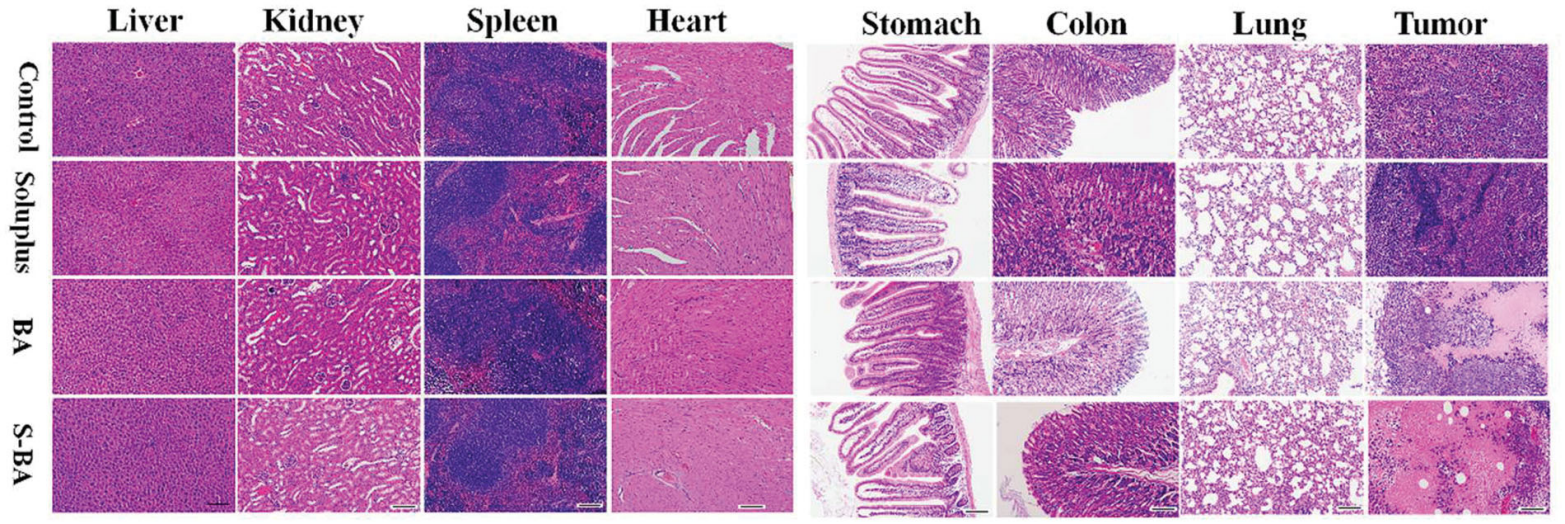
Hematoxylin-eosin (H&E) staining analysis morphological of visceral tissues (Bar = 200 µm). S-BA: Soluplus-BA micelles.

Moreover, mice showed tolerance to Soluplus-BA micelles. During the entire administration process, no mice died and their body weight showed no apparent decrease (Figure 9(B)). The results of H&E staining showed that Soluplus-BA did not cause changes in visceral tissue morphology, confirming the biosafety of Soluplus-BA (Figure 10). The above results prove the low side toxicity of Soluplus-BA micelles.

## 4. Conclusion

In conclusion, BA-loaded Soluplus micelles were prepared using a thin film dispersion method and their effects and mechanisms in inhibiting breast cancer proliferation both *in vivo* and *in vitro* were investigated. Soluplus-BA micelles increased the inhibitory effect of BA on the proliferation of breast cancer MDA-MB-231 cells. Mechanism studies showed that Soluplus-BA induced DNA DSBs and induced cell apoptosis by inducing ROS accumulation. Soluplus-BA could also inhibit angiogenesis by inhibiting the tube formation and migration of HUVEC and inhibiting the neovascularization of CAM. Mechanism study indicated that the angiogenesis inhibitory effect of BA may be accomplished by regulating the HIF-1/VEGF-FAK signaling pathway. Overall, this study confirms that Soluplus micelles can be used as a potential drug delivery system to deliver BA and exert anti-breast cancer effects through multiple mechanisms.

## Supplementary Material

Supplemental Material
