# Supplementary material for: Improved anticancer activity of betulinic acid on breast cancer through a grafted copolymer-based micelles system
Source: Drug Deliv. 2021 Sep 25;28(1):1962–71. doi: 10.1080/10717544.2021.1979125 (PMC8475105; doi:10.1080/10717544.2021.1979125)
Supplement: Supplemental Material [file IDRD_A_1979125_SM0342.docx]

**Improved anticancer activity of betulinic acid on breast cancer through a grafted copolymer-based micelles system**

Xueju Qi^a^, Cong Gao^b,^ ^1^, Chuanjin Yin^a^, Junting Fan^c^ Xiaochen Wu^a^ and Chuanlong Guo^a,^ *

^a^ Department of Pharmacy, College of Chemical Engineering, Qingdao University of Science and Technology, Qingdao 266042, China

^b^ The Third Affiliated Hospital of Shandong First Medical University, Affiliated Hospital of Shandong Academy of Medical Sciences, Jinan 250031, China

^c^ Department of Pharmaceutical Analysis, School of Pharmacy, Nanjing Medical University, Nanjing, 211166, China

^1^ X. Q. and C. G. contributed equally to this work.

*Corresponding author:

Department of Pharmacy, College of Chemical Engineering, Qingdao University of Science and Technology, Qingdao 266042, China. wxcguest@126.com (X C. W.) [gcl_cpu@126.com](mailto:gcl_cpu@126.com) (C L. G.)

**Results**


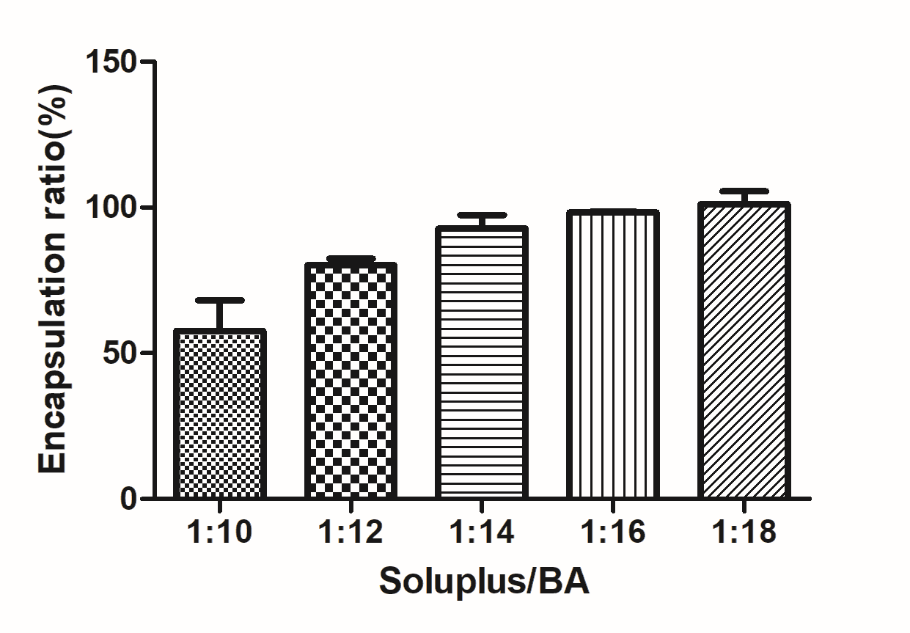


Figure S1 Encapsulation ratio with different Soluplus/BA weight ratio.


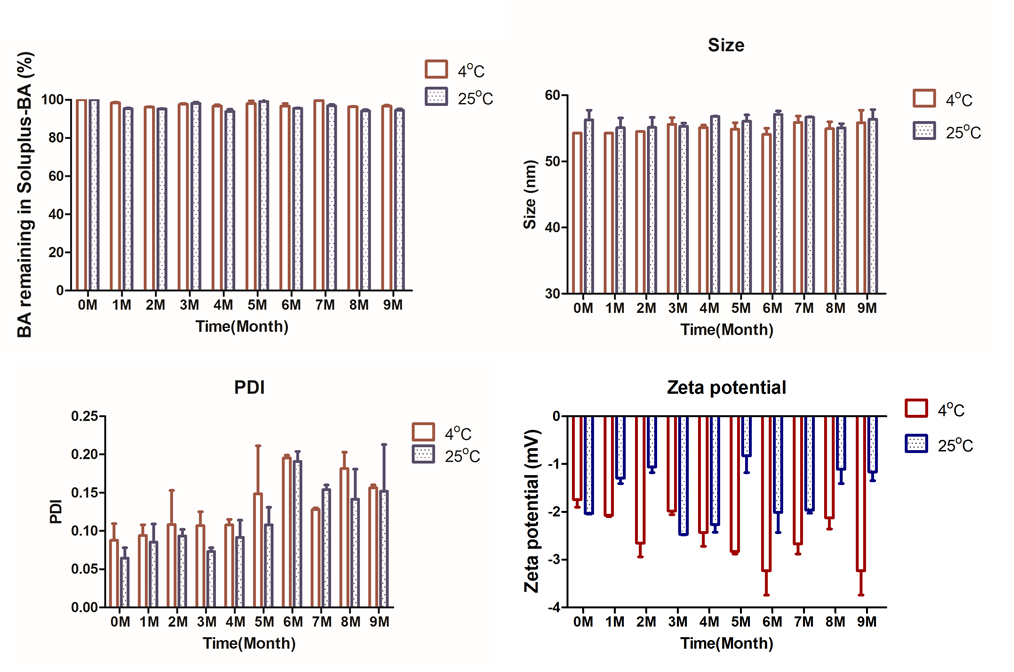


Figure S2 Stability of Soluplus-BA micelles. Soluplus-BA micelles were stored at 25 °C and 4 °C for 9 months.


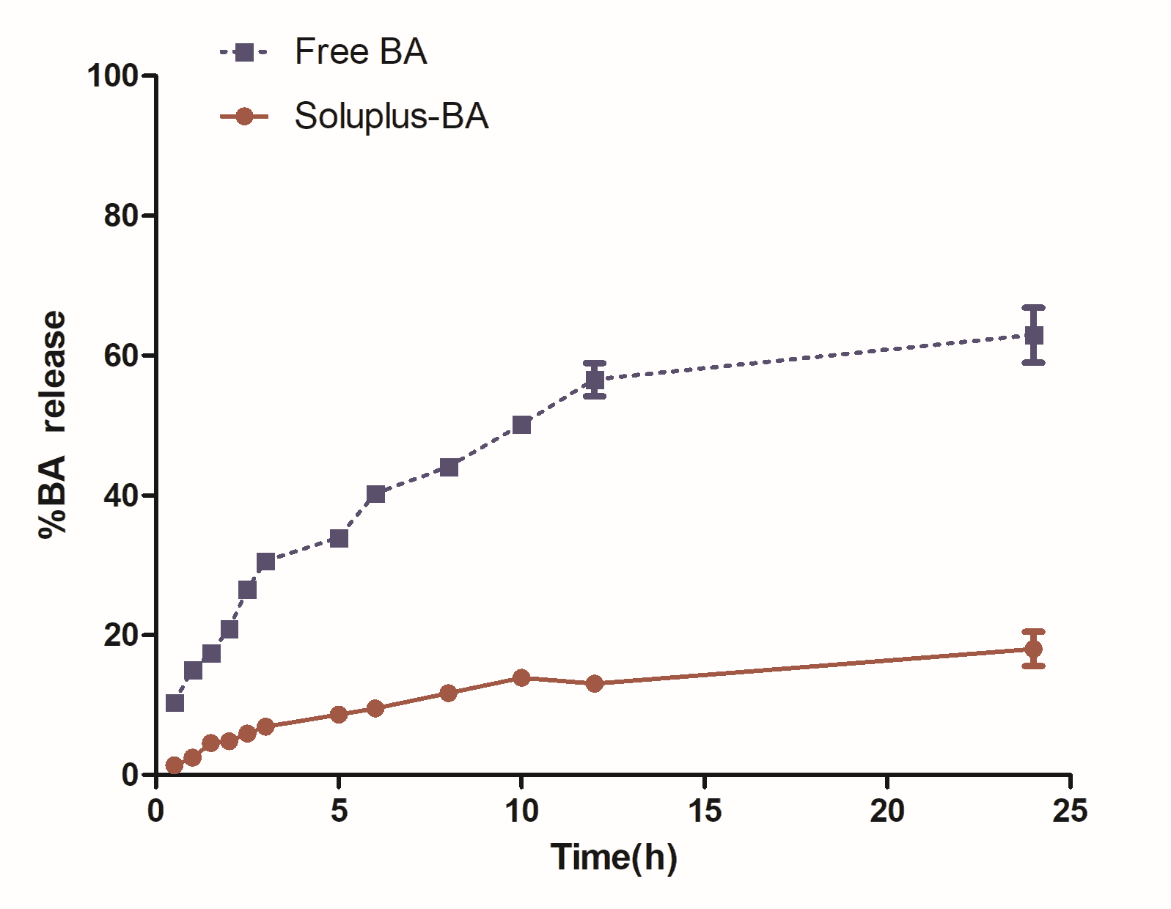


Figure S3 Cumulative drug release profiles of BA from Soluplus-BA.
